# Immunotherapeutic Targeting in Autoimmune Diseases

**DOI:** 10.1155/2016/1432702

**Published:** 2016-12-15

**Authors:** Nona Janikashvili, Maxime Samson, Eli Magen, Tinatin Chikovani

**Affiliations:** ^1^Tbilisi State Medical University, Tbilisi, Georgia; ^2^University Hospital of Dijon, Université Bourgogne Franche-Comté, Dijon, France; ^3^Barzilai University Medical Center, Ben-Gurion University of the Negev, Ashkelon, Israel

The induction and maintenance of immune tolerance represent major therapeutic goals in autoimmunity. Current strategies for controlling autoimmune disorders are based on the administration of immunosuppressive drugs leading to severe infections or resulting in patient relapse following drug withdrawal. More targeted approaches are therefore needed in this context. The administration of monoclonal antibodies against specific inflammatory mediators has been tested as a more refined strategy. Such monoclonal antibodies have highlighted potential in preclinical and clinical applications. Nevertheless, as they target global immune activation pathways, the impairment of regulatory immune responses has also been demonstrated. Therefore, the sustained clinical responses upon their long-term administration are still under debate. Immunosuppressive/regulatory immune cell-based therapy is a relatively recent approach with promising potential. To date, however, the implementation of autologous immunosuppressive cells in the clinic has been limited by their peculiarly low frequency in patients with inflammatory conditions. Strategies aimed at promoting their expansion and enhancing their suppressive function may also open new therapeutic options.

In this special issue, authors addressed topics related to the immune targets of autoimmune processes and seek to identify novel strategies for the therapeutic intervention of cellular, molecular, and genomic instabilities in various autoimmune disorders.

In their case report, A. Pozdzik et al. evaluated the circulating B cell subtypes including plasmablasts (CD3^−^CD19^+^CD20^−^IgD^−^CD27^high^CD38^high^) and memory (CD3^−^CD19^+^CD20^+^IgD^−^CD27^+^CD38^−^) and naive (CD3^−^CD19^+^CD20^+^IgD^+^CD27^−^CD38^low^) B cells in a patient with anti-phospholipase A2 receptor 1 autoantibody (PLA2R1 Ab) related membranous nephropathy (MN) during 4 years of follow-up after rituximab therapy (RTX). The authors suggested that circulating plasmablasts could be a new cellular biomarker of residual autoimmunity in PLA2R1 related MN and will support the rapid assessment of RTX response in clinical practice.

In the line of exploring autoreactive B cell functions, Q. Pan et al. investigated the association between IgG4 autoantibody and complement abnormalities in systemic lupus erythematosus (SLE). The experimental subjects included 72 newly diagnosed and untreated SLE patients, 67 rheumatoid arthritis (RA) patients, and 41 healthy donors, who served as control subjects. The authors concluded that the IgG4 autoantibody (antinuclear IgG4) may dampen the inflammatory response in SLE by competitively binding to autoantigens to form nonpathogenic ICs that result from the low affinity of IgG4 for both the Fc*γ* receptor and the C1 complement molecule, thus maybe providing a novel therapeutic target for SLE.

The complex mechanism of RA involves numerous cell types: lymphocytes, monocytes, and fibroblast-like synoviocytes (FLSs), which, upon activation, produce high levels of proinflammatory cytokines, such as IL-6, TNF-*α*, and IL-1*β*. Of these proinflammatory mediators, IL-6 plays a crucial role as it triggers the hepatic acute-phase response and augments joint inflammation and bone erosion in RA. Inhibition of IL-6 signaling significantly improves autoimmune arthritis in experimental animal models and in RA patients. X. Luo et al. identified Krüppel-like factor 4 (KLF4) to be a TNF-*α*-induced transcription factor that is higher expressed in synovial tissues and fibroblast-like synoviocytes from RA patients than from osteoarthritis patients. These investigators established the notion that KLF4 is localized in the nuclei of RA FLSs and regulates the expression of IL-6 through both direct promoter activation and interaction with NF-*κ*B.

Y. Liu and G. Sun explored the antiarthritic effect of* Periploca forrestii* saponin (PFS) and its derived Periplocin in adjuvant-induced arthritis in rats. The study suggests the antiarthritic activity of PFS and Periplocin via modulation of the key proinflammatory cytokines IL-6, Th1 (IFN-*γ*), Th2 (TGF-*β*1 and IL-13), and Th17 (IL-22) and transcription factor T-bet, GATA3, and C-Jun genes.

M. Ciechomska and S. O'Reilly further summarized the latest information about the potential therapeutic application of epigenetic modifications in targeting immune abnormalities of rheumatic diseases. By covering extensive volume of works and discussing major categories of epigenetic layers and players, these authors highlighted the relevance of the epigenome targeting treatment. While such drugs have already being applied in cancer and cardiovascular diseases, M. Ciechomska and S. O'Reilly addressed the evident emergence of such treatments for autoimmune rheumatic diseases. In another review article, R. Sujashvili overviewed the advantages of extracellular ubiquitin as a new tool for targeted therapy for immune mediated disorders of various etiologies.

In the context of acute inflammation, some enzymes acquire robust proinflammatory activities. A research paper by G. Zhou et al. addressed the role of a lipid-signaling enzyme phospholipase D2 (PLD2) in the pathogenesis of inflammatory bowel diseases (IBD). The authors reported on the high expression of PLD2 in peripheral blood cells and in inflamed mucosa of patients with active IBD. Among various proinflammatory cytokines establishing IBD pathogenic microenvironment, TNF-*α* markedly supported the upregulated expressions of PLD2. Inhibition of PLD2 was associated with the amelioration of the intestinal colitis via promoting neutrophil migration through CXCR2 upregulation. Collectively, these data identified PLD2 as a new therapeutic target for the management of IBD.

This special issue encompasses fundamental and translational data aimed at identifying novel immunotherapeutic targets in autoimmune diseases. We believe that these original research articles and review papers will stimulate the continuing efforts to improve current therapies of patients with such diseases.

